# Identification of polymorphisms in cancer patients that differentially affect survival with age

**DOI:** 10.18632/aging.101305

**Published:** 2017-10-20

**Authors:** Aoife Doherty, Yelena Kernogitski, Alexander M. Kulminski, João Pedro de Magalhães

**Affiliations:** ^1^ Integrative Genomics of Ageing Group, Institute of Ageing and Chronic Disease, University of Liverpool, Liverpool, L7 8TX, United Kingdom; ^2^ Biodemography of Aging Research Unit (BARU), Social Science Research Institute, Duke University, Durham, NC 27708, USA

**Keywords:** ageing, genetics, geriatric oncology, SNP, longevity, WRN

## Abstract

The World Health Organization predicts that the proportion of the world's population over 60 will almost double from 12% to 22% between 2015 and 2050. Ageing is the biggest risk factor for cancer, which is a leading cause of deaths worldwide. Unfortunately, research describing how genetic variants affect cancer progression commonly neglects to account for the ageing process. Herein is the first systematic analysis that combines a large longitudinal data set with a targeted candidate gene approach to examine the effect of genetic variation on survival as a function of age in cancer patients. Survival was significantly decreased in individuals with heterozygote or rare homozygote (i.e. variant) genotypes compared to those with a common homozygote genotype (i.e. wild type) for two single nucleotide polymorphisms (rs11574358 and rs4147918), one gene (*SIRT3*) and one pathway (FoxO signalling) in an age-dependent manner. All identified genes and pathways have previously been associated with ageing and cancer. These observations demonstrate that there are ageing-related genetic elements that differentially affect mortality in cancer patients in an age-dependent manner. Understanding the genetic determinants affecting prognosis differently with age will be invaluable to develop age-specific prognostic biomarkers and personalized therapies that may improve clinical outcomes for older individuals.

## INTRODUCTION

Cancer is a leading cause of death worldwide; approximately 14.1 million new cancer cases and 8.2 million cancer-related deaths were recorded globally in 2012 [1]. Ageing is the biggest risk factor for cancer, and the majority of tumours are diagnosed in patients older than 60 years [2,3]. The World Health Organization predicts that the proportion of the world's population over the age of 60 will almost double from 12% to 22% between 2015 and 2050 (World Health Organization, 2015). The evolving age demography affects cancer incidence and mortality rates, which has serious consequences for a country's healthcare system and economy [4,5]. Novel insights into the age-related genetic predisposition of cancer survival would be a major breakthrough in expanding healthy life span in humans.

The last decade has seen extensive efforts to catalogue human genetic variation [6,7] and to correlate variation with phenotypic traits. For example, single nucleotide polymorphisms (SNPs) have been assessed for statistical associations with complex traits such as longevity [8–14] and various common diseases [15–22]. Case-control studies that compare population SNP frequency to disease characteristics often simply list a set of SNPs statistically significantly associated with a particular condition, not accounting for the ageing process. Unfortunately, this is an oversight, as molecular systems affected by such genetic variants are evolving entities whose interactions change with age [23,24]. For example, the ageing process affects multiple inter-linked molecular systems including the immune [3,25,26], metabolic [27,28] and cardiovascular [29] systems.

Ageing affects cancer incidence rates [30,31], prognosis [32–34] and drug response [35]. Recently, Kulminski et al. (2014) investigated the effects of the e4 allele of the *APOE* gene on human survival in a range of ages from mid-life until extreme old age, and the sensitivity of those effects to cardiovascular disease, cancer and neurodegenerative disease [12]. This allele is thought to have a protective effect against early life infectious disease such as diarrhea and liver damage caused by Hepatitis C virus infection. Their research suggested that, although there is an advantage to the allele in early life, there is a significant adverse effect of the e4 allele on survival that is limited to women with a moderate lifespan (70-95 years). Furthermore, non-skin cancer increased the risk of death of e4 carriers two-fold compared to non-e4 carriers among women of moderate lifespans. These observations suggest the existence of age- and gender-sensitive systemic mechanisms linking the e4 allele to lifespan that can non-additively interfere with cancer-related mechanisms. The research described herein combines the availability of a large longitudinal data set with a targeted candidate gene approach to examine the effect of genetic variation on survival as a function of age from a systematic perspective. Insights obtained from this novel investigation are of high biological importance, as understanding the biomarkers and molecular mechanisms that affect cancer prognosis in an age-dependent manner will provide critical information for age-specific patient outcome and relevant assignment to therapies; an area of research that will only become even more important with a greying population.

## RESULTS

### Data set assembly and quality filtering: phenotypic and genotypic data

The population under study in this analysis is the Framingham Heart Study (FHS). In brief, the FHS comprises >10,000 individuals in different cohorts who have been examined every 2-4 years for up to 60 years (depending on cohort; see Methods). The FHS has previously successfully addressed interesting biological questions related to ageing and disease [11,12,37,38].

Using FHS data, two cancer data sets were assembled based on tumour topography for all patients that were first diagnosed with cancer over the age of 50; all except skin cancer (AESC; n=1,194) and all except skin and sex cancer (AESSC; n=867) (Table S1a). Survival was defined as the length of time (in years) between initial diagnosis and death. In this study, all-cause mortality is being considered. Survival data was organised into: full data set (i.e. those diagnosed aged 50+), and three age categories: individuals diagnosed between the ages of 50 and 64, diagnosed 65 and 79, and those diagnosed aged 80 plus. Herein, we focus on the larger data set, AESC; however, the results for AESSC are also described in the supplementary material. Unless otherwise stated, the results for the two data sets were similar, with the exception that the smaller AESSC data set tended to lose statistical power more quickly. In addition to cancer survival data, we also collected information about tumour grade, co-morbidities, sex, cohort and familial relationships for each individual, in order to consider the impact that these factors have on survival (see Methods).

The FHS Affymetrix 50K Human Gene Focused microarray was employed in this study. There were 36,647 SNPs remaining after a quality filtering and linkage disequilibrium (LD) analysis (see Methods). The primary objective of our analysis was to identify polymorphisms and corresponding molecular mechanisms that affect survival in an ageing-related manner. Thus, we used the commonly implemented approach that focuses on SNPs that are in close proximity to known longevity associated genes (LAGs) [42,61–64]. A set of 316 putative longevity associated genes (LAGs) was assembled (Table S1d); and 880 SNPs located near/within 245 of the 316 longevity associated genes were extracted for analysis. The commonly used dominant genotypic model was employed, in which the heterozygous and variant homozygote genotypes were combined into a single risk group (the “variant”/“Var” group) and compared to the common homozygote (the “wild type”/“WT” group) [12,68,69].

Subsequently, we conducted a gene-level analysis by examining the 245 genes that the 880 SNPs that had been assigned to in the previous paragraph (Table S1e). A LAG could have (1) One SNP per gene, (2) Two SNPs per gene or (3) More than two SNPs per gene. For the “one SNP per gene” category, each individual was classified as possessing either the wild type or variant genotype at the SNP position. For the “two SNPs per gene” category, each individual was classified depending on the combination of wild type and variant alleles that they possessed at each SNP position; “WT_WT” (i.e. wild type at first SNP position and wild type at second SNP position) or “WT_Var”, “Var_WT” or “Var_Var”. For the “more than two SNPs per gene” category, individuals were classed as having a low (<33%), medium (34-65%) or high (>66%) number of wild types. Each gene was assigned to an age-related KEGG pathway, where possible. Individuals were described as either having a low (<50%) or high (>50%) number of wild type SNPs per pathway. In total, 880 SNPs were assigned to 245 aging-related genes and 18 pathways.

To identify differences in survival patterns in different genotypes for a set of ageing-related variants between different diagnosis age brackets, we conducted a survival analysis, comprising (1) Kaplan Meier (KM) Estimator and (2) Cox Proportional Hazards Model. A Kaplan Meier Curve constructs a survival curve to compare the survival patterns of two or more groups of individuals, and a Log Rank Test is subsequently implemented to examine the null hypothesis that there is no difference between the populations in the probability of an event (in this case, death) occurring at a time point. A Kaplan Meier Curve and Log Rank Test cannot account for any other possible confounding factors that may affect survival, and so a Cox Proportional Hazard Regression Model subsequently modelled survival as a function of other variables, including genotype, sex, comorbidity status, cancer grade and cohort. Data sets were then divided by sex, cohort, and equal numbers of genotypes to examine the robustness of both the Kaplan Meier and Cox Model analyses. The reader is directed to the exact number of individuals used in each analysis at relevant times throughout the manuscript.

There is one SNP (rs11574359), two genes (*GPX4* and *SIRT3*) and one pathway (FoxO) that demonstrated age-related patterns of survival in cancer patients and will be discussed in turn. For each SNP, gene and pathway of interest, the results are laid out as a description of: (1) Kaplan Meier Curve, (2) Kaplan Meier Curve using sub-sets of the data (i.e. using equal numbers of genotypes, and dividing the data set by sex and cohort), (3) Cox Model and (4) Cox Model results once the model is adjusted for co-variates of interest rather than stratified, and once the data set is split by sex and cohort.

### Rs11574358, a non-synonymous SNP in the WRN gene, has an age-dependent impact on mortality in cancer patients

A Kaplan Meier analysis was conducted for each of the 880 SNPs assigned to LAGs (Table S2a for full output from Kaplan Meier analysis for all SNPs). Four SNPs consistently demonstrate significant (FDR < 0.05) survival differences between the wild type and variant genotypes in different age categories (Table S2b): rs1794108, rs4147918, rs11574358 and rs317913. In all of these cases, possessing the wild type confers a longer survival time. In this section, we will focus on one SNP, rs11574358, the only SNP whose survival differences between genotypes in an age-dependent manner remained significant after the implementation of the Cox Model. However, it is interesting that the other SNPs significant from the Kaplan Meier analysis (i.e. rs1794108, rs4147918 and rs317913) are all located within well-known cancer and ageing related genes. For example, rs1794108 is a missense deleterious mutation in the proteasome 26S subunit, non-ATPase 13 (*PSMD13*) that is involved in cellular senescence [85], ageing [86] and with the onset of various cancers [87–89]. *ABCA7*, the gene that rs4147918 is located within, is on chromosome 19p13.3, the same chromosomal section as *APOE*, a gene that is well known to be associated with ageing and longevity related traits [21,90,91]. *ABCA7* has also been implicated recently in cancer progression; [92,93]; the SNP itself has also been previously associated with Alzheimer's Disease [94]. Finally, rs317913 is located within the ral guanine nucleotide dissociation stimulator-like 3 gene (*RGL3*). Both the SNP [95,96] and the gene have previously been identified with cancer-related traits [97].

**Figure 1:**
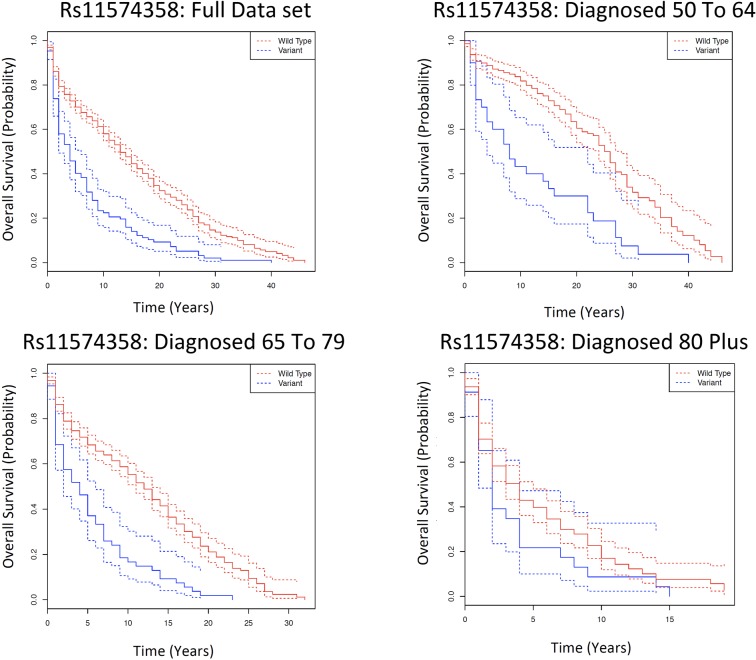
Kaplan Meier survival estimates of overall cancer survival for rs11574358 among the Framingham Heart Study according to a dominant genotype model for different age categories, in which the wild type is the dominant homozygote, and the variant is the heterozygote and the minor homozygote. The full data set indicates all individuals diagnosed with cancer over the age of 50; and subsequently each age category is the individuals diagnosed with cancer in that particular age category. Solid lines indicate survival curve, dashed line indicates 95% confidence interval.

In the Kaplan Meier analysis; for rs11574358, in the full data set (i.e. those diagnosed 50+), the WT genotype patients have a significantly longer 5-year survival rate (5YSR) than those with the variant (Var) genotype (5YSR: WT: 70.1% (95% CI: 67.2-72.9%), Var: 40.1% (95% CI: 31.8-50.6%), FDR= 3.45E-12). Similar significant survival differences are observed in those diagnosed 50 to 64 (FDR= 3.0E-4) and diagnosed 65 to 79 (FDR= 1.16E-7), but not in the diagnosed 80 plus age bracket (FDR= 0.85) (Fig. 1, Table S2b).

A number of analysis repetitions were conducted to ensure the robustness of the observations; these are described in further detail in the Methods. First, in each of the age categories, an equal number of wild type and variant genotypes were randomly extracted, and the analysis was repeated, statistically significant survival patterns (FDR < 0.01) observed in all age groups except those individuals diagnosed over the age of 80 (Table S3a1). However, in this case, although the number of individuals is constant between the genotypes within each age category, there are still different numbers of individuals between the age categories. Thus, for the larger AESC data set, 20 individuals were subsequently elected at random for each genotype for all age categories, and the analysis was repeated; this ensures that the number of individuals remains constant per genotype both within and between all age categories. The Log Rank P Values are significant different in both those diagnosed 50 to 64 (P=3.94E-03) and those diagnosed 65 to 79 (P=2.41E-03) (Table S3a). Dividing the AESC data set up by sex and cohort, statistically significant (FDR <0.05) survival differences are still observed in all the age categories except those diagnosed over 80 in the offspring (Table S3c) cohort, and in males (Table S3d) and females (Table S3e); and in the full data set and those diagnosed 65 to 79 in the original cohort (Table 3b). Thus, we suggest that the differences in survival patterns between the wild type and variant genotypes between the different age categories cannot be easily explained by the effects of cohort, sex or sample size.

The genotypic and phenotypic distribution of the data set used in the Cox Model is found in Table S3f. After the Cox Model, that accounted for the effects of sex, cohort, comorbidities, tumour grade at diagnosis and familial relationships, different survival patterns are still observed in all of the age categories except those diagnosed over the age of 80. The hazard ratio of the risk allele in the full data set (i.e. diagnosed 50+) is 1.95 (95% CI: 1.47-2.58; FDR= 2.64E-05) indicating that those individuals with the variant genotype (i.e. heterozygous or rare homozygote) have a 1.95-fold increased risk of death compared to those that possess the wild type (common homozygote). The HR increases to 4.65 (95% CI: 2.56-8.43; FDR= 6.01E-06) in the diagnosed 50 to 64 age category, and decreases to 2.81 (95% CI: 1.77-4.46; FDR= 2.57E-04) for those diagnosed 65 to 79 (Table 1, Table S3g). There are no significant survival differences in those individuals diagnosed >80 (FDR= 0.98). The Cox Model analysis was repeated using slightly varying data sets to ensure robustness of observations; as described in the Methods. First, the same significant age-dependent effects on survival as described are observed by adjusting the Cox model co-variates instead of stratifying (Table S3h). Second, significant or marginally significant survival differences in the same age categories are still observed once the data set is separated by sex (Females: Table S3i; Males: Table S3j) and offspring cohort (Table S3l) and in those diagnosed 65 to 79 in the original cohort (Table S3k), or if an equal number of individuals (AESC: *N*=176; AESSC: *N*=119) are randomly selected from each age category and the analysis is repeated (Table S8a).

**Table 1:** Summary of the significant SNP, genes and pathways of interest after the Cox model analysis

Analysis	WT Allele (HR = 1.0)	Risk Allele	Gene	Age Category	N in KM analysis	HR	95% CI	P Value	FDR
**SNP Analysis**									
**rs11574358**	**TT**	**GT+GG**	***WRN***	**50+**	1,133	1.95	1.47-2.58	2.83E-06	2.64E-05
				**50-64**	378	4.65	2.56-8.43	6.40E-02	6.01E-06
				**65-79**	558	2.81	1.77-4.46	8.65E-01	2.57E-04
				**80+**	197	1.16	0.56-2.17	0.27	0.98
**Gene Analysis**									
***GPX4***	**WT1_WT2**	**Var1_WT2**	***GPX4***	**50+**	1,035	1.25	0.94-1.47	0.12	0.48
				**50-64**	351	1.32	0.54-3.22	0.53	0.82
				**65-79**	504	1.85	1.85 (1.24-2.74)	2.3E-3	0.02
				**80+**	180	1.40	0.72-2.69	0.32	0.74
***SIRT3***	**High # WT**	**Low # WT**	***SIRT3***	**50+**	1,037	1.49	1.06-2.03	0.02	0.09
				**50-64**	353	1.23	0.59-2.56	0.57	0.81
				**65-79**	501	1.98	1.21-3.21	0.007	0.06
				**80+**	183	1.02	0.51-2.01	0.96	0.96
**Pathway Analysis**									
**FoxO Pathway**	**High # WT**	**Low # WT**	-	**50+**	1,146	1.13	0.85-1.51	0.39	0.39
				**50-64**	382	1.31	0.72-2.38	0.37	0.39
				**65-79**	564	1.90	1.13-3.11	0.014	0.058
				**80+**	200	1.52	0.69-3.37	0.29	0.39

Column “N in KM Analysis” is the number of individuals in the original Kaplan Meier Curve. The number of individuals per genotype in the Cox Model is found in supplementary material. Column 5, “HR”, is the hazard ratio of the risk allele relative to the wild type. 95% CI is the 95% confidence interval of this HR. *P* value represents the significance of the HR and FDR is the FDR (Benjamini-Hochberg)-corrected *P* value.

Rs11574358 is a non-synonymous SNP in the Werner (*WRN*) gene converting a serine to an alanine. SIFT, a sequence homology-based tool that predicts deleterious substitutions based on the degree of conservation of amino acid residues based on alignments of closely related sequences [98] predicts that this variant is deleterious to protein function.

### Rs4147918 in *GPX4*, and *SIRT3*, display patterns of age-dependent differences in mortality

**Figure 2:**
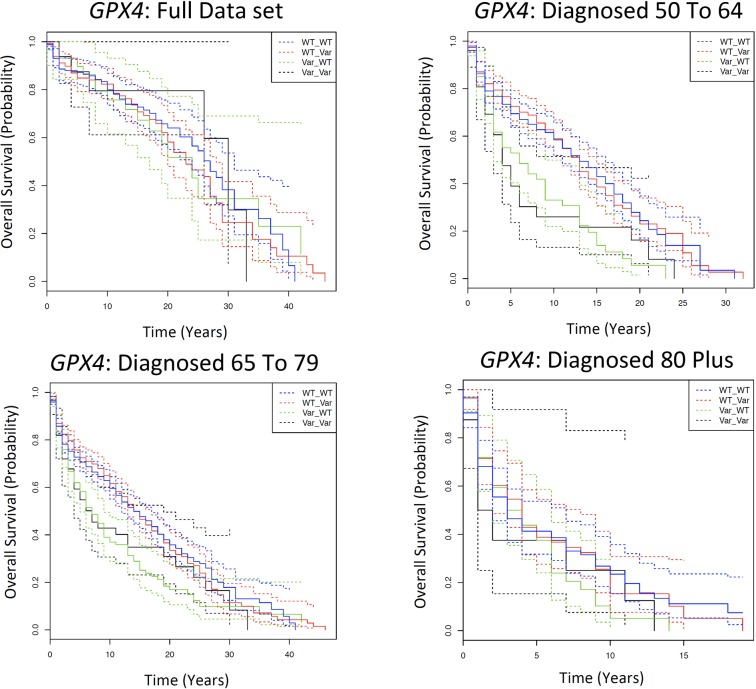
Kaplan Meier survival estimates of overall survival for *GPX4* among the Framingham Heart Study according to a dominant genotype model, in which the wild type is the dominant homozygote, and the variant is the heterozygote and the minor homozygote. The full data set indicates all individuals diagnosed with cancer over the age of 50; and subsequently each age category is the individuals diagnosed with cancer in that particular age category. Solid lines indicate survival curve, dashed line indicates 95% confidence interval.

Each of the 880 SNPs in the analysis was assigned to a gene ±60kb of the SNP. There are 60 genes with one SNP assigned to the gene, 54 genes with two SNPs assigned to the gene, and 131 genes with more than two SNPs assigned to the gene (Table S1f). The full set of Kaplan Meier results for the gene analysis is in Table S2c. There was one gene of interest with two SNPs per gene; glutathione peroxidase 4 (*GPX4*) that had two SNPs assigned to it: rs4147918 and rs757232. In the KM analysis, there are significantly different survival differences between the genotypes in both the full data set (FDR= 0.046) and in those diagnosed 65 to 79 (FDR= 0.013). In these two age categories, the average 5YSR for those with a WT allele in the first SNP position (i.e. a WT_WT or WT_Var genotype) is at least 17% higher than those with a variant allele in the first position (i.e. a Var_WT or Var_Var genotype; Fig. 2, Table S2d). There are no statistically significant differences in survival between genotypes for those diagnosed 50 to 64, or those diagnosed 80 plus (FDR=> 0.05). To examine the effects of sample size on the observations, 20 individuals were subsequently randomly selected from each of the genotypes per age category and the analysis was repeated (Table S4a). Similar to earlier observations, there are no statistically different survival patterns between the genotypes in any of the age categories except those diagnosed 65 to 79 (P Val = 0.03). Similar observations were made once the data set is divided by sex (Males: Table S4b, Females: Table S4c) and in the original cohort once the data is divided by cohort (Table S4d) but not in the offspring cohort (Table S4e).

The genotypic and phenotypic distribution of the data set used in the Cox Model is in Table S4f. In the “diagnosed 65 to 79” age category, possessing the “Var_WT” genotype led 1.85-fold increased risk of death compared to the “WT_WT” genotype (95% CI=1.24-2.74; FDR = 0.02). In all other age categories of the AESC data set, there was no statistically significant difference in survival between genotypes (Table S4g, Table 1). A similar pattern is observed when variables are adjusted for rather than stratified in the Cox model (Table S4h) and pre-FDR significance is observed if the data set is divided by sex (Males: Table S4i, Females: Table S4j) and in the Original cohort (Table S4k), but not the Offspring cohort (Table S4l). A similar observation of age-dependent survival differences between allele combinations with marginal significance is made in both the AESC (N=347) and the AESSC (N=287) data sets if an equal number of individuals is randomly selected from all age categories; Table S8b). Thus, the age dependent effects of this gene are not easily explainable by sample size, cohort, sex, familial relationship or tumour characteristic effects.

The data suggests that having a variant allele at the first position (rs4147918) and a wild type allele at the second position (rs757232) leads to an increased risk of death for patients with cancer in an age-dependent manner. SNPs were allowed to be ±60kb of a gene in this data set. Thus, although the SNPs were technically assigned to *GPX4*, rs4147918 (i.e. the variant) is located in the nearby *ABCA7* gene, while rs757232 (the wild type) is in Histocompatibility (Minor) HA-1 gene (*HMHA1*). Rs757232 did not display significantly different survival patterns after the KM analysis in the individual SNP analysis (Table S2a). However, rs4147918 significantly affects survival in an age-dependent pattern in the SNP-level KM analysis (Full Data set FDR = 1.54E-04, Diagnosed 65 to 79= 9.51E-06; Table S2b) and is located within a known cancer and ageing related gene, as described in the previous section.

**Figure 3:**
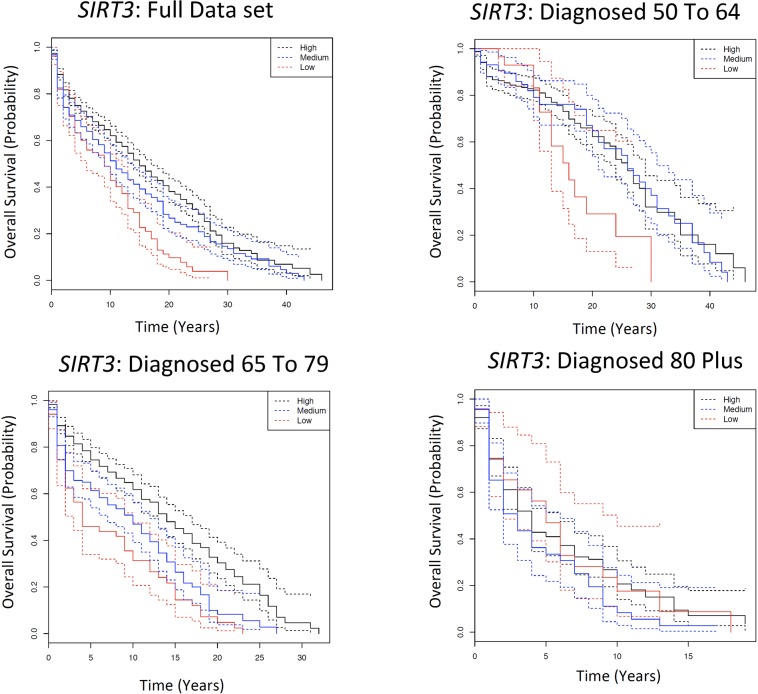
Kaplan Meier survival estimates of overall survival for *SIRT3* among the Framingham Heart Study according to a dominant genotype model, comparing patients with a high number of wild types to those with a low number of wild types. The full data set indicates all individuals diagnosed with cancer over the age of 50; and subsequently each age category is the individuals diagnosed with cancer in that particular age category. Solid lines indicate survival curve, dashed line indicates 95% confidence interval.

A second gene of interest is NAD-dependent deacetylase sirtuin-3, mitochondrial (*SIRT3*) that was assigned four SNPs: rs11246007, rs11246020, rs1794108 and rs2280544. Each individual is characterised depending on the number of wild type alleles: “low” (one), “medium” (two) or “high” (three or four) number of wild type alleles. In *SIRT3*, possessing a high number of wild types led to at least 20% longer 5YSR in both the full data set (FDR=8.8E-07) and those diagnosed 65 to 79 (FDR=8.8E-07; Fig. 3, Table S2e).

Similar to previous sections, the effects of sample size were considered by randomly selecting 20 individuals from both wild type and variant genotypes for each of the age categories and repeating the analysis, thus ensuring that the number of individuals per genotype is constant both within and between all age categories. In agreement with our observations, there were no statistically significant survival differences in any of the age categories except marginal significance for those diagnosed between the age of 65 and 79 (Log Rank P Val = 0.09; Table S5a). Similar age-dependent survival differences between genotypes for this gene are observed if the data set was divided by cohort (Original Cohort: Table S5b, Offspring Cohort: Table S5c) or sex (Males: Table S5d, Females: Table S5e).

The genotypic and phenotypic distribution of the data set used in the Cox Model is found in Table S5e. After conducting a Cox Model analysis, individuals with a low number of wild types that were diagnosed 65 to 79 have a 1.98-fold (95% CI=1.21-3.21; FDR= 0.06) increased risk of death compared to those with a high number of wild type alleles (Table S5g). A similar observation are consistently observed if one adjusts for the co-variates rather than stratifies (Table S5h), and similar significant or pre-FDR significant or marginally significant (in the case of original cohort) pattern is observed once the data is split by cohort or sex (Tables S5i-S5l). Similar patterns are found with marginal significance in the AESSC data set if an equal number of individuals (*N*= 119) is selected from each age category and the analysis is repeated; and in both AESC (*N*=347) and AESSC (*N*=287) data sets if equal numbers of individuals are randomly selected from the full data set, those diagnosed 50 to 64 and those diagnosed 65 to 79 age categories (Table S8).

SNPs were allowed to be ±60kb of a gene in this data set. Rs11246007 is an intron variant in *SIRT3.* Rs11246020 is a missense mutation in *SIRT3* that converts a valine to an isoleucine. Rs2280544 is a UTR Variant 3′ SNP in the BET1 Golgi Vesicular Membrane Trafficking Protein Like (*BET1L*) gene. Interestingly, rs1794108 is one of four SNPs that consistently demonstrated survival differences in the initial Kaplan Meier SNP analysis, as described earlier (Table S2a).

### SNPs in the FoxO pathway display age-dependent patterns of mortality

**Figure 4:**
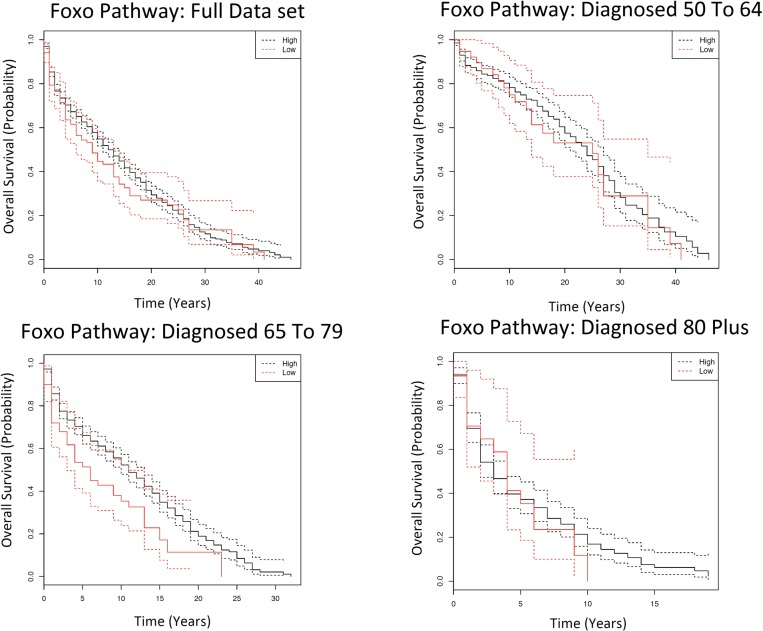
Kaplan Meier survival estimates of overall survival for FoxO pathway among the Framingham Heart Study according to a dominant genotype model, comparing patients with a high number of wild types to those with a low number of wild types. The full data set indicates all individuals diagnosed with cancer over the age of 50; and subsequently each age category is the individuals diagnosed with cancer in that particular age category. Solid lines indicate survival curve, dashed line indicates 95% confidence interval.

Six of the eighteen putative ageing-related pathways (Rap1 signalling, FoxO signalling, Cell Cycle, p53 signalling, Fc epsilon signalling and TNF signalling) displayed marginally significant (FDR < 0.10) survival differences in the KM analysis between two genotype groups (i.e. having a low and high number of wild types) in different age groups (Table S2f). Un-surprisingly, these pathways are known to be involved in cancer-related processes; e.g. p53 signalling, Cell Cycle and Rap1 signalling (Table S2f). There is one pathway of interest after the Cox Model that will be discussed in detail: FoxO signalling. There 29 genes (Table S6a) and 108 SNPs (Table S6b) assigned to the FoxO pathway. In the KM analysis, there is significantly different survival patterns observed in those diagnosed 65 to 79 (FDR=0.058; Fig. 4, Table S2g). In this case, having a high number of wild type alleles confers a protective effect on survival. Unlike all of the other SNPs and genes, the same pattern is not replicated once equal numbers individuals per genotype per age category are extracted and the Kaplan Meier analysis is repeated (Table S6c). In the diagnosed 65 to 79 age category, significantly different survival patterns between genotypes are observed in the original cohort (Table S6d) and in females (Table S6g) but not in the offspring cohort (Table S6e) or in males (Table S6f).

The genotypic and phenotypic distribution of the data set used in the Cox Model is in Table S6h. After the Cox model, possessing a low number of wild types leads to 1.90 (95% CI: 1.13-3.11; FDR=0.058) increased risk of death compared to possessing a high number of wild types in those diagnosed 65 to 79 (Table S6i; Table 1). Subsequently, a similarly significant increased hazard ratio is observed for those with a low number of wild type alleles in the diagnosed 65 to 79 age category if the Cox model is adjusted for co-variates rather than stratified (Table S6j), and similar pre-FDR significance is observed if the analysis is conducted on each sex separately (Table S6k, S6l) and in original cohort (Table S6m), but not in the offspring cohort (Table S6n). Post-FDR significantly different survival is also observed in the AESC (N=349) and AESSC (N=139) data sets if an equal number of individuals are selected from each age category and the analysis is repeated (Table S8d). The three most common types of the effects for the SNPs in the FoxO pathway are intron variants (38%), non-coding transcript variants (17%) and mis-sense variants (11%).

### Replication of results in an independent population

A replication population was selected based on the combination of two cohorts; Atherosclerosis Risk in Communities Study and Cardiovascular Health Study; the assembly of these data sets is described in detail in the methods. We attempted to replicate our results in this combined replication population; however the Framingham Heart Study is quite unique; both in terms of the large amount of data collected over a long period of time and the SNPs that were genotyped on its unique custom Human Gene Focussed Affy 50K array. Unfortunately, although this replication population has been invaluable in the past in investigating various traits associated with ageing and longevity (for example, [12,99]), there was a low overlap in the SNPs (16 SNPs) out of all of the SNPs of interest in the SNP/gene/path-way analysis in this study and the ARIC/CHS arrays. Only two SNPs of interest could be considered further in our analysis; however, rs11574358; a key SNP of interest in our analysis had a minor allele frequency of 0 in both the ARIC and CHS data sets and so could not be considered further. Thus, the replication study purely focussed on rs4147918 (MAF of 0.036 and 0.044 in CHS and ARIC, respectively).

For rs4147918, 92% (N=1,808) of the data set was assigned as wild type (i.e. homozygous dominant for this polymorphism), while 8% (N=140) were con-sidered as variants. The cancer phenotype available for this study in ARIC/CHS included cancers at all sites because there was no information on cancer without skin in ARIC. Comorbidity phenotype was defined as the score counting presence of the following diseases in an individual (each disease was coded as 1; no disease was coded as 0): heart failure, diabetes, stroke, and/or myocardial infarction. A Kaplan Meier analysis indicated that there were significantly different survival patterns only in the Full Data set (P=0.03) and not in any of the specific age categories, although the difference was marginally significant in those diagnosed 65 to 79 (P=0.06; Table S7). Thus, there were no significant results of note after the Cox Model and the relationship between survival between the different age categories could not be considered using this population (Table S7).

## DISCUSSION

The investigation described herein is the first systematic study to address how the ageing process impacts the effect that single nucleotide polymorphisms have on cancer survival; a field that could greatly affect the possibility of individualising cancer prognoses and treatments in the post-genomic era. There were two SNPs (rs11574358 and rs4147918), one gene (*SIRT3*) and one pathway (FoxO signalling) that may be of interest for further consideration.

Rs11574358 is a non-synonymous SNP in the Werner (*WRN*) gene. *WRN* is responsible for the progeroid Werner Syndrome, characterised by the accelerated appearance of features associated with ageing [100–102]. This syndrome is well established to be associated with an elevated risk of cancer [103,104]. Some of the most common co-occurring chronic co-morbidities among cancer patients include ischemic heart disease, hypertension and hyperlipidemia [58]. Rs11574358 was recently identified to be associated with traits related to ageing, including cardiovascular disease prevalence, systolic blood pressure, cancer prevalence, total cholesterol and cystatin C in serum (chronic kidney disease) [42,105]. Since we know that rs11574358 is associated with traits such as cardiovascular disease prevalence and systolic blood pressure and the polymorphism converts a serine to an alanine residue, and has been suggested to abolish the phosphoserine structure of the protein and potentially affect protein function [106]; it is possible that this SNP may exert an age-dependent effect on mortality by contributing to the effect or severity of the most common cancer co-morbidities. Alternative hypotheses could be that the polymorphism may be associated with cancer aggressiveness, tissue susceptibility to cancer invasion or to changes in other ageing processes that contribute to mortality.

The second SNP of interest was rs4147918; this SNP was assigned to the *GPX4* LAG in this analysis, but as SNPs were allowed to be ±60kb of a LAG, the SNP is actually located within the nearby *ABCA7* (Entrez ID: 10347), a member of the superfamily of ATP-binding cassette transporters that transport various molecules across extra- and intra-cellular membranes. Rs4147918 induces a glutamine-to-arginine change in an exon of the *ABCA7* gene. Although this SNP is considered tolerated (i.e. not considered deleterious to function), even a variant that can be tolerated, or a synonymous change that appears not to affect amino acid selection at all, could still affect protein function and disease susceptibility, particularly when combined with the ageing process [107–110]. *ABCA7* is on chromosome 19p13.3, the same chromosomal section as *APOE*, a gene that is well known to be associated with ageing and longevity related traits [12,21,91,111,112]. In *ABCA7*, seven SNPs (including rs4147918) are cholesterol-related and showed a significant association with Late Onset Alzheimer's Disease [21], although little else is reported on the clinical effects caused by this polymorphism. Thus, it is possible that similar to rs11574358, if some of the most common chronic co-morbidities among cancer patients include ischemic heart disease, hypertension and hyperlipidemia [58]; and rs4147918 is known to be cholesterol-related and associated with the ageing process, then it is possible that this polymorphism may exert an effect on age-dependent mortality by changing the severity of particular co-morbidities.

There was one gene of interest for further analysis; *SIRT3,* with four SNPs (although the SNPs are in *SIRT3*, and two closely-related genes *PSMD13* and *BET1L*). Interestingly, *SIRT3* is known to be involved in both the cancer and ageing processes [113–115]. In addition, *PSMD13* and *SIRT3* share a promoter [116], and *PSMD13*, a proteasome subunit, is involved in the degradation of abnormal proteins, cellular senescence [85] and ageing [86] and variants in this gene have previously been associated with the onset of various cancers [87–89]. Thus, it is plausible that having a high number of variants in closely related genes could impact cancer progression in an age-dependent manner.

There was one pathway of interest for potential further investigation; FoxO signalling. The ability of FoxO factors to induce cell cycle arrest, DNA repair and apoptosis makes them attractive candidates as tumour suppressors [117,118]. In addition, members of the FoxO pathway are known to affect ageing and longevity [117–119]. Thus, since having a high number of variants appears to negatively affect cancer survival and the pathway is known to play an important role in the aetiology of cancer and the ageing process, perhaps a high level of variation in this pathway affects mortality in an age-dependent manner; which should be considered further.

There are a number of limitations to this investigation. First, it was not possible to identify an ideal replication population, as the Framingham Human Gene Focussed Affy 50K array is a unique custom array. We did not consider cancer-specific deaths in this instance; due to lack of data (In the AESC full data set, there were 663 causes of deaths recorded, 47 of these had cancer as a primary cause of death). Although all-cause mortality is a common end-point for many successful survival analyses [18,49,50,55], we acknowledge that the absence of data on cancer-specific deaths versus all other causes of death limits our ability to interpret our observations, and leads to more questions. It would be interesting in the future to do similar investigations with cancer-specific death data available, in order to tease apart the specific contribution that variants such as those described in this research are contributing to specifically cancer survival.

As with all polymorphism-trait studies, it is possible that our SNPs of interest do not exert effects on mortality themselves, but are in high linkage disequilibrium with the ungenotyped SNPs of interest. Some of the effects observed in this analysis would appear to be quite modest; for example, the gene and pathway analyses exhibit modest effects (∼2 fold increased risk of death in particular age groups). However, similarly-sized effects have been reported in other analyses which have provided fascinating insight into the impact of genetic variants on various survival patterns [12,18,74,120,121]. In addition, given the complex nature of longevity and ageing as traits and the known difficulties in identifying SNPs and genes associated with these traits in even much larger human studies that do not account for the ageing process [122], the identification of SNPs that display even modest effects on cancer survival differently with age warrant further attention. Finally, it is not clear why age-dependent survival effects are generally observed in those diagnosed 65 to 79, rarely in those diagnosed 50 to 64 and never in those diagnosed over the age of 80. To eliminate potential bias caused by unequal sample size, the Kaplan Meier curves were repeated, using the same number of individuals per genotype between and within age categories, and the Cox Model analyses were divided by sex and cohort. One potential reason for a lack of observation in diagnosed >80 group could be that the overall mortality rate in the older population is such that more individuals die of unrelated causes, and so die with cancer but not due to cancer. However, this does not explain the lack of observation in the diagnosed 50 to 64 group. In addition, Kulminski et al. (2014) similarly demonstrated that there is a significant adverse effect of the e4 allele on survival that is limited to women with a moderate lifespan (70-95 years); i.e. an effect was also not observed in the young (<70 years) or extremely old (>95 years) age categories. They suggested that possible reasons for these observations could include the buffering mechanisms by other genes [123] and/or the environmental modulations of genetic effects [124]. Therefore, an interesting open question arising from this work is why some variants affect survival in certain age groups, but not in others.

In summary, this investigation suggests that the *APOE* variant identified by Kulminski et al. [12] may not be the sole variant that affects cancer survival in an age-dependent manner. This study is the first exploratory systematic investigation to identify SNPs, genes and pathways that differentially affect mortality depending on the age of diagnosis, whose findings need to be independently validated in a suitable population, once such a population arises in the future. If corroborated, such information would provide potential targets for further exploration as prognostic biomarkers and individualised therapies in the post-genomic era. Given that we live in a greying population, and the majority of tumours are diagnosed in aged patients, such knowledge will be an invaluable tool advancing the field of geriatric oncology.

## MATERIALS AND METHODS

### Study population: the Framingham Heart Study

The Framingham Heart Study (FHS) [36] comprises 5,209 respondents aged 28-62 at baseline who have been biennially examined for almost 60 years. The Framingham Heart Study Offspring (FHSO) respondents (N=5,124) aged 5-70 at baseline were biological descendants (N=3,514), their spouses (N=1,576) and adopted offspring (N=34) of the FHS participants who were examined about every four years at nine visits. The study design has been detailed previously [39,40]. Phenotypic data was collected through FHS clinic examinations, hospital admission surveillances and monitoring death registries. Biospecimens were mostly collected in the late 1980s and through the 1990s from surviving participants [12]. The FHS data are available from the NIH SHARe through dbGaP [41] (accession number phs000007.v29.p10).

### Assembly of cancer and comorbidity phenotypic data set

As described in the Results, two cancer data sets were assembled based on tumour topography for all patients that were first diagnosed with cancer over the age of 50 and were not diagnosed solely via death cert: (1) All cancer except skin cancer (AESC; n=1,194) and (2) All cancer except skin and sex cancer (AESSC; n=867). Skin cancer is commonly not considered in similar analyses (for example, [12,42]) due to accurate diagnosis difficulties that may affect survival times [43–48]. Sex-related cancers were subsequently also removed to examine survival differences from cancers that are common to both sexes, as conducted in [12]. All-cause mortality is being considered in this analysis, this has been a common end-point for many successful survival analyses [17,49,50]. The data was right-censored for the Kaplan Meier analysis. If date of death was not available, the date of last contact was used.

Factors that may affect survival were considered. Comorbidities (i.e. additional diseases that co-occur with a primary disease of interest) can affect cancer diagnosis [51], treatment [52–54] and prognosis [55–57]. The co-morbidities included in our analysis were: cerebrovascular disease, diabetes, congestive heart failure, dementia, myocardial infarction and subsequent cancer diagnoses; some of which are the most common co-morbidities in cancer [2,58,59]. Each individual was classified as having a low (0-2), medium (3-4) or high (5-6) number of co-morbidities. A tumour grade (1-4) was also assigned to each tumour.

### Assembly and quality filtering of the genetic data set

The FHS Affymetrix 50K Human Gene Focused microarray was employed in this study. Quality control filtering was conducted as in [42] using PLINK v. 1.9 [60]: SNPs were removed if they exhibited Hardy Weinberg *P* values <10^−2^, >10% missingness, >2% Mendel errors, <2% Minor Allele Frequency (MAF) or were located on a sex chromosome. Using PLINK, a LD analysis was also conducted between SNPs by calculating pair-wise r^2^ statistics for founders only, SNPs in high LD (i.e. r^2^ > 0.9) were removed.

A set of 298 human LAGs (http://genomics.senescence.info/genes/human.html; Table S1b) and 83 human homolog to mouse genes (http://genomics.senescence.info/genes/models.html; Table S1c) were obtained from GenAge Build 17 [65]. Once the two data sets (i.e. human, and human homolog to mouse) were combined and redundant genes were removed, 316 putative LAGs remained (Table S1d). The location of each gene was retrieved from Ensembl [66], and SNPs located within ±60kb of each putative LAG were extracted, similar to [42,67]. 880 SNPs were putatively identified as ageing-related using this method, covering 245 of the 316 genes in the data set (Table S1e). SNPs were subsequently assigned to the 245 LAGs; as described in the Results, a LAG could have one, two or more than two SNPs assigned to it. Third, SNPs were assigned to LAG pathways. A set of 69 pathways considered related to ageing process given their known involvement in ageing related processes were extracted from the KEGG database v. 74 [70] and 18 pathways that had at least 10 genes and 10% overlap between the pathway genes and the set of longevity associated genes in this analysis were extracted [70] (Table S1g).

### Statistical analyses: the Kaplan Meier Estimator and Cox Proportional Hazards Model

A KM curve was constructed for each SNP/gene/pathway of interest using the “survival” package v. 2.38 [71] in R v. 3.2.2 [72]. A Log Rank Test [73] subsequently examined the null hypothesis that there is no difference between the populations in the probability of an event (in this case, death) occurring at a time point. Robustness of the constructed Kaplan Meier curves were examined by repeating the analysis twice: (1) Using equal number of wild type and variant genotypes and (2) Once the data set was divided by cohort (i.e. original and offspring cohorts) and sex (i.e. males and females).

The KM Estimator and Log Rank Test do not allow other explanatory variables to be considered when estimating the survival differences between two groups. Thus, for SNPs, genes and pathways that displayed initial survival differences using the KM approach, a Cox Proportional Hazard Regression Model modelled survival as a function of genotype, sex, comorbidity status, cancer grade and cohort, implemented in the “coxme” package v. 2.2.3 [71] in R v. 3.2.2 [72]. Schoenfield residuals were used to test whether the proportional hazards assumption of the model were met, and only models with *P* values >0.05 were considered valid. As a result of non-proportionality, the models were stratified by sex, comorbidity status, cancer grade and cohort, similar to what has been conducted in other studies [74–76]. Genotypes were treated as categorical variables. In addition, a multivariate kinship frailty model implemented in the kinship package (http://cran.r-project.org/web/packages/kinship/) in R was incorporated to account for any familial relationships within the FHS [39,77]. Missing grade and genotypic information was imputed with the widely used multiple imputation method [78–81], implemented in MICE v. 2.25 [82]. For the Cox Model, in the SNP analysis, the hazard ratio (HR) describes the increase (or decrease) risk of death in the Var allele, compared to the WT allele. In the gene-level analysis, the risk of each genotype group is being compared to either the WT_WT allele (for those genes with two SNPs) or possessing a high number of WT alleles (for those genes with >2 SNPs). In the pathway analysis, the risk of each genotype group is being compared to those individuals with a high number of WT alleles.

To examine the robustness of the Cox Model, we repeated the analysis twice: (1) By adjusting the model for all of the factors instead of stratification (i.e. cohort, sex, grade, co-morbidities and, for the SNP analysis, other SNPs of interest from the Kaplan Meier analysis) and (2) Once the data was split by sex (i.e. male and female) and cohort (i.e. original and offspring), and using an equal number of individuals per genotype and age category. A Benjamini-Hochberg False Discovery Rate (FDR) was calculated across the AESC and AESSC data sets, all age brackets and all SNPs, genes or pathways of interest. In addition, once the data was divided based on cohort (i.e. original and offspring) or sex, the FDR was conducted across all conditions (i.e. male, female, original and offspring). A FDR <0.05 was considered statistically significant.

### Replication analysis in an independent population

A replication population was selected based on the combination of two cohorts. In the Atherosclerosis Risk in Communities Study (ARIC) [83], the study participants (aged 45-64 at baseline in 1987) were randomly selected and recruited at four field centres across the U.S. We used data from four available examinations. Genotyping in 12,771 ARIC participants (N=9,633 Caucasians) was conducted using Affymetrix 6.0 array (1,000K SNPs). For the Cardiovascular Health Study (CHS) [84], the main cohort of the CHS participants (N=5,201 Caucasians) aged 65+ years at baseline in 1989 was examined annually through 1999. The CHS clinic exams ended in June 1999. After June 1999 two phone calls per year to participants collected information on incidence of diseases and death. Deaths also were ascertained through surveillance and at semi-annual contacts. SNPs for the present study were selected from the Candidate Gene Association Resource (CARe) that included records for 5,531 CHS participants. This investigation combined the two studies to carry out our analyses, restricting to partici-pants of European descent.

### Availability of data and materials

The data generated during this study (for example survival study results) is available in the supplementary material. The Framingham SHARe data used for the analyses described in this manuscript were obtained through dbGaP (accession number phs000007.v29.p10). The URL for this study is: https://www.ncbi.nlm.nih.gov/projects/gap/cgi-bin/study.cgi?study_id=phs000007.v29.p10&hv=159482&phd=1105&pha=4313&pht=1415&phvf=&phdf=&phaf=&phtf=&dssp=1&consent=&temp=1.

### Ethics approval and consent to participate

FHS data was obtained from the Framingham Heart Study of the National Heart Lung and Blood Institute of the National Institutes of Health and Boston University School of Medicine. This work was supported by the National Heart, Lung and Blood Institute's Framingham Heart Study (Contract No.N01-HC-25195). For this type of study, formal consent is not required.

## SUPPLEMENTARY MATERIAL TABLES
















